# A Rare Case of Amyand’s Hernia

**DOI:** 10.7759/cureus.38641

**Published:** 2023-05-06

**Authors:** Siddharth Shah, ShahZeib Syed, Keenan Bayrakdar, Omar Elsayed, Kavya Poluru

**Affiliations:** 1 Internal Medicine, Lewis Gale Medical Center, Salem, USA; 2 Internal Medicine, LewisGale Medical Center, Salem, USA

**Keywords:** surgical case reports, rare case report, rare complication of appendicitis, symptomatic hernia, amyands hernia

## Abstract

A hernia is an abnormal protrusion of an organ or tissue from its containing cavity. The most common type of abdominal hernia is an inguinal hernia. When a hernia is non-reducible, it is termed an incarcerated hernia. We present one such rare case of an incarcerated appendix within a right inguinal hernia, also called Amyand’s hernia (AH). We discuss current approaches toward surgically repairing this type of complicated hernia and a complication that can arise if it is not repaired in a timely manner.

## Introduction

A hernia is defined as an abnormal protrusion of an organ or tissue from its containing cavity. Inguinal hernia repair remains one of the most commonly performed surgeries worldwide and this type of hernia accounts for 80% of abdominal wall hernias [1]. An incarcerated hernia is non-reducible and strangulated hernia means its blood supply is compromised, leading to ischemia and necrosis [1,2]. Almost one-third of males are diagnosed with an inguinal hernia during their lifetime. It is most commonly seen in adults over 50 years of age. Only 3% of women will develop an inguinal hernia. In the United States, the annual incidence of inguinal hernia is 315 per 100,000, and surgical repair of inguinal hernias accounted for more than 48 billion dollars in healthcare expenditures in 2005 [3].

We report a case of a 70-year-old man who presented with diffuse abdominal pain and was found to have a non-reducible, incarcerated Amyand’s hernia (AH). The patient underwent robotic right inguinal hernia repair with mesh. AH is repaired only via surgery, which has both diagnostic and therapeutic roles. This is a rare case because only 1% of all hernias contain the appendix [1,2].

## Case presentation

The patient was a 70-year-old male who presented to the outpatient surgery appointment with diffuse abdominal pain rated as 7/10. The patient himself is a physician and had undergone a robotic cholecystectomy three years prior, and understood that he had an inguinal hernia warranting a surgeon’s evaluation. On physical exam, the abdomen was nondistended and symmetrical, bowel sounds were present in all four quadrants, and a right inguinal hernia was noted. The patient was scheduled for surgery one month after his outpatient appointment.

Per the operative report, the patient was laid supine, with both arms up, prepped, and draped in a sterile fashion using chlorhexidine. Time-out was done to identify the correct patient and correct procedure. All present in the room were in agreement. Access was gained supraumbilically with a Veress needle. Pneumoperitoneum to 15 mmHg was achieved with carbon dioxide. Under the right upper and left upper quadrants, an 8-mm trocar was placed. A preperitoneal flap was created right at the arcuate line. The space of Retzius was dissected laterally, and the inguinal space was dissected. There was an indirect hernia with incarcerated noninflamed appendix. The sac was easily dissected off the vas all the way to the genu of the vas. The myopectineal view of safety was achieved, and an extra-large preformed Bard lightweight mesh was placed without difficulty. Robotic right inguinal hernia repair with mesh was performed successfully due to the postoperative diagnosis of incarcerated right inguinal hernia.

Two weeks after surgery, the patient presented for his follow-up appointment. He endorsed stabbing right lower quadrant pain rated as 6/10 one day postoperatively that had resolved after Tylenol use as needed for two to three days. On physical exam, the abdomen was non-tender on light palpation with normal bowel sounds. Moreover, well-healing incisions showing no evidence of infection were noted. The patient was explained that the pain was likely due to inflammation postoperatively. He was instructed to follow up in four weeks again; however, the patient never followed up as he remained asymptomatic.

## Discussion

AH was first described in an 11-year-old patient based on the observation of an appendix in an inguinal hernia with perforation and fecal fistulation [1]. It is an exceedingly rare condition estimated to impact only about 1% of all inguinal hernia cases [4]. This disease tends to impact children and males more but can be seen in females at any age as well [4].

We described a case of an elderly male with AH that was managed with robotic right inguinal hernia repair with mesh. The noninflamed appendix was removed from the inguinal sac and mesh was placed to repair this hernia. It has been postulated in previous reports that inflammation would eventually occur with restriction of blood flow to this structure with prolonged herniation. Hence, early intervention has been noted to be crucial in the management of this hernia, as it is associated with notably high rates of morbidity and mortality [5,6].

There is some contention as to whether synthetic mesh plugs should be contraindicated in the setting of AH given the risk of creating a septic nidus or if appendectomy is necessary in all cases. In this case, the appendix was removed from the sac with mesh repair as it was incarcerated. This case provides further evidence that in cases of AH, the appendix should be removed to avoid complications.

## Conclusions

AH is an exceedingly rare surgical condition impacting 1% of all inguinal hernia cases. Early diagnosis and surgery can help prevent lifelong complications like neuropathic pain. Furthermore, a laparoscopic approach is beneficial in reducing the incidence of surgical site infections.
